# Subsequent risk of ipsilateral and contralateral invasive breast cancer after treatment for ductal carcinoma in situ: incidence and the effect of radiotherapy in a population-based cohort of 10,090 women

**DOI:** 10.1007/s10549-016-3973-y

**Published:** 2016-09-08

**Authors:** Lotte E. Elshof, Michael Schaapveld, Marjanka K. Schmidt, Emiel J. Rutgers, Flora E. van Leeuwen, Jelle Wesseling

**Affiliations:** 1 Department of Molecular Pathology, The Netherlands Cancer Institute/Antoni van Leeuwenhoek, Plesmanlaan 121, 1066 CX Amsterdam, The Netherlands; 2 Department of Psychosocial Research and Epidemiology, The Netherlands Cancer Institute/Antoni van Leeuwenhoek, Plesmanlaan 121, 1066 CX Amsterdam, The Netherlands; 3 Department of Surgery, The Netherlands Cancer Institute/Antoni van Leeuwenhoek, Plesmanlaan 121, 1066 CX Amsterdam, The Netherlands; 4 Department of Pathology, The Netherlands Cancer Institute/Antoni van Leeuwenhoek, Plesmanlaan 121, 1066 CX Amsterdam, The Netherlands

**Keywords:** Ductal carcinoma in situ, Invasive breast cancer, Surgery, Radiotherapy, Population-based cohort study

## Abstract

**Purpose:**

To assess the effect of different treatment strategies on the risk of subsequent invasive breast cancer (IBC) in women diagnosed with ductal carcinoma in situ (DCIS).

**Methods:**

Up to 15-year cumulative incidences of ipsilateral IBC (iIBC) and contralateral IBC (cIBC) were assessed among a population-based cohort of 10,090 women treated for DCIS in the Netherlands between 1989 and 2004. Multivariable Cox regression analyses were used to evaluate associations of treatment with iIBC risk.

**Results:**

Fifteen years after DCIS diagnosis, cumulative incidence of iIBC was 1.9 % after mastectomy, 8.8 % after BCS+RT, and 15.4 % after BCS alone. Patients treated with BCS alone had a higher iIBC risk than those treated with BCS+RT during the first 5 years after treatment. This difference was less pronounced for patients <50 years [hazard ratio (HR) 2.11, 95 % confidence interval (CI) 1.35–3.29 for women <50, and HR 4.44, 95 % CI 3.11–6.36 for women ≥50, *P*_*interaction*_ < 0.0001]. Beyond 5 years of follow-up, iIBC risk did not differ between patients treated with BCS+RT or BCS alone for women <50. Cumulative incidence of cIBC at 15 years was 6.4 %, compared to 3.4 % in the general population.

**Conclusions:**

We report an interaction of treatment with age and follow-up period on iIBC risk, indicating that the benefit of RT seems to be smaller among younger women, and stressing the importance of clinical studies with long follow-up. Finally, the low cIBC risk does not justify contralateral prophylactic mastectomies for many women with unilateral DCIS.

**Electronic supplementary material:**

The online version of this article (doi:10.1007/s10549-016-3973-y) contains supplementary material, which is available to authorized users.

## Introduction

Ductal carcinoma in situ (DCIS) is a potential precursor lesion of invasive breast cancer (IBC) [1]. Most women (80–85 %) diagnosed with DCIS present with a mammographic abnormality without clinical symptoms [2]. Since the introduction of population-based mammographic screening and, more recently, digital mammography, the incidence of DCIS has increased substantially [3–7]. In the Netherlands, the European standardized rate of in situ breast carcinoma—of which DCIS is the most common type (~80 %)—increased fivefold since 1989, up to 25.1 per 100,000 women in 2013 [8]. In the United States, the incidence (age adjusted to the 2000 US standard population) increased even more: from 5.8 per 100,000 in 1975 to 33.8 per 100,000 women in 2010 [9].

The natural course of DCIS is not well known because DCIS has almost always been treated by mastectomy or breast-conserving surgery (BCS) with or without radiotherapy (RT). Between 1988 and 2011, only 2 % of women with DCIS were managed without surgery in the United States [10]. In the Netherlands, the percentage of non-operated DCIS between 1989 and 2004 was 0.8 % [11].

Women with DCIS are treated to prevent the development of IBC, assuming that this may lead to a reduction in breast cancer-specific deaths. Some women with unilateral DCIS even undergo contralateral prophylactic mastectomy. However, the long-term benefit of treating asymptomatic DCIS that may or may not progress to IBC is difficult to quantify [12]. Therefore, screening programs are criticized to be associated with overdiagnosis and resultant overtreatment of DCIS [13, 14].

Considerable uncertainty remains about the likelihood that a treatment strategy will prevent IBC, whether that likelihood will change based on specific patient and DCIS characteristics, and whether the reduction in risk is enough to justify the costs and the potential side effects of that treatment [12]. The effect of different treatment strategies on the risk of subsequent events in women diagnosed with DCIS has been addressed previously in both prospective trials and observational studies [15–27]. However, many of these studies focused on local recurrences, not discriminating between invasive and non-invasive events, or did not have complete information on treatment. Moreover, several studies have analyzed specific subgroups, such as “favorable” and “good-risk” DCIS, or focused on a specific treatment strategy.

Gierisch et al. prioritized research needs for DCIS patients, and pointed out the assessment of the effect of treatment strategies on IBC, using existing observational data [12]. We assessed the effect of DCIS treatment strategies on risk of subsequent ipsilateral invasive breast cancer (iIBC) using a large population-based cohort with complete information on treatment and follow-up. In addition, we analyzed the risk of contralateral IBC (cIBC).

## Methods

### Patient selection

**Fig. 1:**
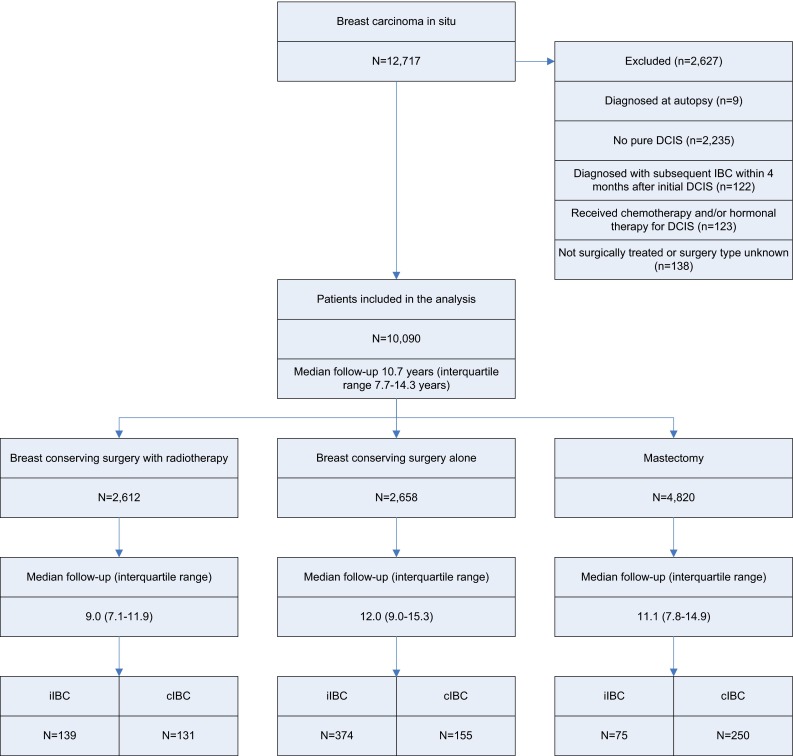
Flow diagram for patient selection and median follow-up by initial treatment type. *iIBC* ipsilateral invasive breast cancer, *cIBC* contralateral invasive breast cancer

All women diagnosed with breast carcinoma in situ in the Netherlands between January 1st 1989 and December 31st 2004 were selected from the Netherlands cancer registry (NCR) managed by the Netherlands Comprehensive Cancer Organization. Patients with previous malignancies, except for non-melanoma skin cancer, were not included. This cohort (*n* = 12,717) was linked to the nationwide network and registry of histology and cytopathology in the Netherlands (PALGA) [28]. The selection criteria for this study were a diagnosis of pure DCIS, i.e., no lobular or other subtype component, and only treated by surgery with or without RT. See Fig. 1 for a detailed list of the excluded cases (*n* = 2627). The study was approved by the review boards of the NCR and PALGA.

### DCIS treatment and other characteristics

Information on treatment, age, date of diagnosis, and grade was derived from data provided by NCR. Guidelines for DCIS treatment in the Netherlands recommend mastectomy or BCS, consisting of microscopic complete tumor excision. From 1999, the addition of RT after BCS is included in the recommendation. Adjuvant (hormonal) treatment is not recommended. Primary DCIS treatment was categorized into (1) BCS+RT; (2) BCS alone; and (3) mastectomy. Initial treatment was defined as the final treatment for the ipsilateral breast within 3 months after DCIS diagnosis. For patients for whom surgery type was not coded by NCR, we retrieved this information from PALGA. We validated whether patients registered by NCR as treated with BCS had indeed undergone BCS using the conclusions of pathology reports within 3 months of DCIS diagnosis. Furthermore, we validated surgical treatment for women who developed subsequent iIBC after mastectomy, using conclusion texts of all available pathology reports. Subsequently, we assessed whether women initially treated with BCS had undergone ipsilateral mastectomy during follow-up, using both NCR and PALGA data.

Based on the gradual implementation of the national breast cancer screening program, we categorized year of DCIS diagnosis into two periods: 1989–1998 (implementation phase) and 1999–2004 (full coverage). Age was subdivided into two groups: <50 and ≥50 years. Grade was available for 53 % of the entire cohort. The grading system used in the Netherlands is based on the classification presented by Holland et al. [29].

### Follow-up data

The occurrence of iIBC and cIBC was ascertained based on NCR data, and additionally, for patients treated with BCS, through evaluating pathology reports. Follow-up for subsequent IBC and vital status were complete until at least January 1, 2011.

### Statistical analyses

Time at risk started at date of DCIS diagnosis and stopped at date of diagnosis of the event of interest (iIBC or cIBC), date of death or emigration, or January 1, 2011, whichever came first. We calculated cumulative incidence of iIBC and cIBC using death as competing risk. *P* values were based on competing risk regression [30], with time since DCIS diagnosis as time-scale and adjusted for age (continuous). Further, we compared cumulative incidence of cIBC with the expected cumulative incidence of IBC in the general population. Expected cumulative incidence was derived from age- and period-specific cancer incidence and overall mortality in the Dutch female population, estimated using the conditional method [31].

Cox proportional hazards analyses, using age as primary time-scale and time since DCIS diagnosis as secondary time-scale (0–5, 5–10, and ≥10 years), were used to quantify the effects of different treatments on iIBC and cIBC risks. Period of DCIS diagnosis and age group at DCIS diagnosis were added as covariables. Proportional hazard assumptions were verified using graphical and residual-based methods.

To examine whether iIBC risk differed by grade, we performed a subgroup analysis for women with a reported grade. Because the proportion of women with missing data on grade was more than 30 % up to 1998, we performed this subgroup analysis for women diagnosed between 1999 and 2004.

Surgical treatment was either analyzed as initial DCIS treatment (cumulative incidence) or as a time-varying variable including subsequent mastectomies (Cox regression analysis).

All statistical analyses were performed using STATA/SE 13.1 (StataCorp LP, College Station, TX). A two-sided *P* value less than 0.05 was considered statistically significant.

## Results

### Patient characteristics

Analyses included 10,090 women (Fig. 1), of whom 7931 (79 %) women were ≥50 years at DCIS diagnosis. Median age at DCIS diagnosis was 57.6 years (interquartile range 50.7–66.3 years). Median follow-up was 10.7 years (interquartile range 7.7–14.3 years). During follow-up, 1856 patients died. Table 1 shows characteristics, events and follow-up of the study population by treatment group.

**Table 1:** Characteristics of the study population by treatment group

Number of DCIS patients (%)				
Initial DCIS treatment	BCS+RT	BCS alone	Mastectomy	Total
Age at DCIS diagnosis, years, median (interquartile range)	57.2 (51.2–65.2)	58.9 (51.2–67.2)	57.1 (49.9–66.5)	57.6 (50.7–66.3)
Age at DCIS diagnosis (years)				
<40	91 (3.5)	108 (4.1)	360 (7.5)	559 (5.5)
40–49	367 (14.1)	371 (14.0)	862 (17.9)	1600 (15.9)
50–59	1087 (41.6)	942 (35.4)	1553 (32.2)	3582 (35.5)
60–69	739 (28.3)	785 (29.5)	1245 (25.8)	2769 (27.4)
70–79	308 (11.8)	335 (12.6)	630 (13.1)	630 (13.1)
>80	20 (0.8)	117 (4.4)	170 (3.5)	170 (3.5)
Period of DCIS diagnosis				
1989–1998 (implementation phase)	751 (28.8)	1677 (63.1)	2603 (54.0)	5031 (49.9)
1999–2004 (full nationwide coverage)	1861 (71.3)	981 (36.9)	2217 (46.0)	5059 (50.1)
DCIS grade (1999–2004^a^)				
1	215 (13.6)	302 (40.8)	190 (10.2)	707 (16.9)
2	578 (36.7)	235 (31.7)	554 (29.6)	1367 (32.6)
3	783 (49.7)	204 (27.5)	1128 (60.3)	2115 (50.5)
Subsequent ipsilateral mastectomy				
No	2497 (95.6)	2345 (88.2)	NA	9662 (95.8)
Yes	115 (4.4)	313 (11.8)	NA	428 (4.2)
Follow-up interval, years, median (interquartile range)	9.0 (7.1–11.9)	12.0 (9.0–15.3)	11.1 (7.8–14.9)	10.7 (7.7–14.3)
Follow-up interval (years)				
0–4^b^	101 (3.9)	202 (7.6)	301 (6.2)	604 (6.0)
5–9	1458 (55.8)	656 (24.7)	1741 (36.1)	3855 (38.2)
≥10	1053 (40.3)	1800 (67.7)	2778 (57.6)	5631 (55.8)
Subsequent invasive breast cancer^c^				
No	2351 (90.0)	2167 (81.5)	4501 (93.4)	9019 (89.4)
Ipsilateral only	130 (5.0)	336 (12.6)	68 (1.4)	534 (5.3)
Contralateral only	122 (4.7)	117 (4.4)	243 (5.0)	482 (4.8)
Ipsilateral+contralateral	9 (0.3)	38 (1.4)	7 (0.15)	54 (0.5)
Total	**2612**	**2658**	**4820**	**10090**

*BCS* breast-conserving surgery, *RT* radiotherapy

^a^Data on grade is presented for cases diagnosed from 1999. Grade was not reported in 870 women (17.2 %)

^b^Nine patients with follow-up time = 0 (BCS+RT *n* = 1, BCS alone *n* = 2, Mastectomy = 6)

^c^One patient with unknown laterality of subsequent invasive breast cancer

### DCIS treatment

**Fig. 2:**
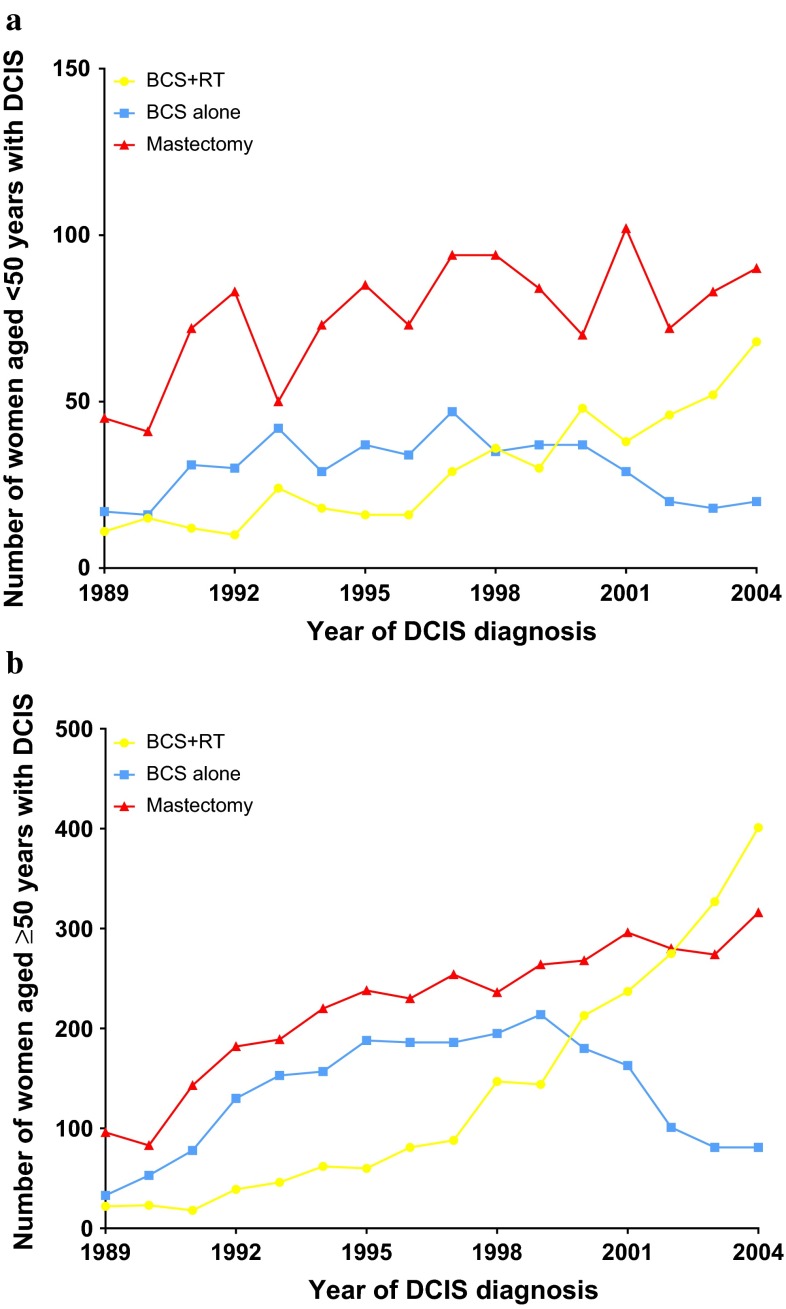
Treatment strategy by year of diagnosis for **a** women <50 years and **b** women ≥50 years. *BCS* breast-conserving surgery, *RT* radiotherapy

Nearly 48 % (*n* = 4820) of DCIS patients were initially treated with mastectomy. Of all 5270 women initially treated with BCS, 50 % additionally received RT. Use of BCS increased over time in women <50 years (*P*_*trend*_ = 0.010) and ≥50 years (*P*_*trend*_ < 0.001). The use of RT after BCS also increased over time in both groups (*P*_*trend*_ < 0.001) (Fig. 2). Fifteen years after initial DCIS treatment, cumulative incidence of subsequent ipsilateral mastectomy was 5.2 % in the BCS+RT group, versus 12.0 % in the BCS-alone group.

### Ipsilateral invasive breast cancer

During follow-up, 588 women developed an iIBC. The median time to iIBC was 5.8 years (interquartile range 2.8–9.0 years). Fifteen years after DCIS diagnosis, cumulative incidence of iIBC was 1.9 % [95 % confidence interval (95 % CI) 1.5–2.4 %] after mastectomy, 8.8 % (95 % CI 7.1–10.8 %) after BCS+RT, and 15.4 % (95 % CI 13.9–17.0 %) after BCS alone.

When assessing the risk of iIBC by treatment, the proportional hazards assumption was violated. We accounted for time dependency in the treatment effect by addition of an interaction term that involved time and treatment to the model (*P*_*interaction*_ < 0.001). Additionally, we found that the effect of treatment was different depending on age group (*P*_*interaction*_ < 0.0001). An extra interaction term that involved period of diagnosis and treatment was not significant (*P*_*interaction*_ = 0.445). Therefore, Table 2 presents the effect of treatment on iIBC risk by follow-up interval and age group.

**Table 2:** Multivariate Cox regression analysis for iIBC in women treated for DCIS

Age group at DCIS diagnosis	Follow-up time	Treatment	Total iIBC	Person-time (years)	HR (95 % CI)	*P* value
<50 years	0–5 years	BCS+RT	17	2186	Ref	
		BCS alone	36	2108	2.11 (1.35–3.29)	0.001
		Mastectomy	19	6237	0.35 (0.20–0.61)	<0.001
	5–10 years	BCS+RT	19	1579	Ref	
		BCS alone	23	1668	1.01 (0.66–1.55)	0.95
		Mastectomy	12	5414	0.13 (0.07–0.23)	<0.001
	>10 years	BCS+RT	15	808	Ref	
		BCS alone	20	1346	0.78 (0.46–1.33)	0.37
		Mastectomy	11	4455	0.20 (0.11–0.37)	<0.001
≥50 years	0–5 years	BCS+RT	29	10394	Ref	
		BCS alone	141	9542	4.44 (3.11–6.36)	<0.001
		Mastectomy	15	18066	0.27 (0.16–0.46)	< 0.001
	5–10 years	BCS+RT	48	6971	Ref	
		BCS alone	112	7077	2.13 (1.54–2.96)	<0.001
		Mastectomy	9	14806	0.10 (0.06–0.17)	<0.001
	>10 years	BCS+RT	11	2353	Ref	
		BCS alone	40	4391	1.64 (1.01–2.69)	0.05
		Mastectomy	11	9515	0.15 (0.08–0.29)	<0.001
Period of DCIS diagnosis						
1989–1998			412	67011	Ref	
1999–2004			176	41906	0.72 (0.59–0.87)	0.03
Age group at DCIS diagnosis						
<50 years			172	25801	Ref	
≥50 years			416	83116	0.38 (0.25–0.59)	<0.001

With age as primary time-scale, and treatment as time-varying variable

*iIBC* ipsilateral invasive breast cancer, *HR* hazard ratio, *CI* confidence interval, *BCS* breast-conserving surgery, *RT* radiotherapy

**Fig. 3:**
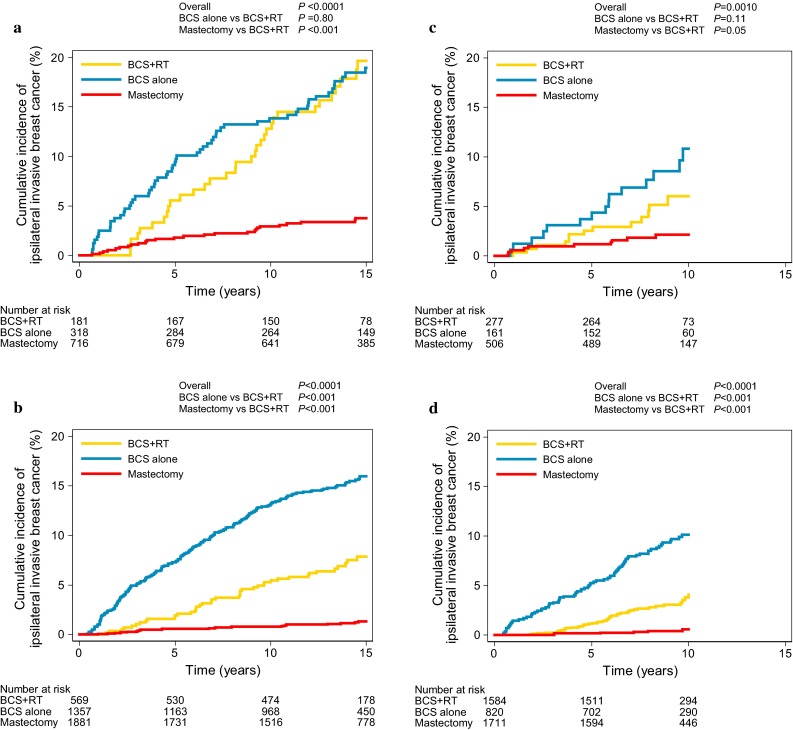
Cumulative incidence of iIBC by treatment strategy for **a** women <50 years diagnosed between 1989 and 1998 **b** women ≥50 years diagnosed between 1989 and 1998 **c** women <50 years diagnosed between 1999 and 2004 **d** women ≥50 years diagnosed between 1999 and 2004, with death as competing risk. *BCS* breast-conserving surgery, *RT* radiotherapy. *P* values based on competing risk regression, adjusted for age (continuous) [30]

Women diagnosed with DCIS between 1999 and 2004 were less likely to develop iIBC than women diagnosed between 1989 and 1998, regardless of treatment and age [hazard ratio (HR) 0.72, 95 % CI 0.59–0.87]. After adjusting for treatment and period, women ≥50 years had lower iIBC risk than <50 women years (HR 0.38, 95 % CI 0.25–0.59). Figure 3 shows the cumulative incidence of iIBC by treatment strategy stratified by period of DCIS diagnosis and age group at DCIS diagnosis.

Both women <50 and ≥50 years treated with BCS alone had a higher risk of developing iIBC than women treated with BCS+RT in the first 5 years after DCIS treatment. However, for women ≥50 years, the difference in iIBC risk after BCS alone compared to BCS+RT was much larger than for women <50 years (HR 2.11, 95 % CI 1.35–3.29 for women <50 years and HR 4.44, 95 % CI 3.11–6.36 for women ≥50 years). While among patients <50 years at DCIS diagnosis, risk of iIBC no longer differed after 5 years following BCS+RT or BCS alone (HR 1.01, 95 % CI 0.66–1.55 for 5–10 years follow-up and HR 0.78, 95 % CI 0.46–1.33 for ≥10 years follow-up), for women ≥50 years, iIBC risk remained increased after BCS alone during subsequent follow-up intervals, although the difference in risks was smaller than in the first 5 years (HR 1.64, 95 % CI 1.01–2.69 for ≥10 years follow-up). A trend in the proportional reduction with age was found when the data were subdivided into three groups according to age: <45, 45–55, and >55 years (data not shown).

Women undergoing mastectomy were less likely to develop iIBC compared to women undergoing BCS (Table 2). The highest absolute iIBC risk after mastectomy was seen for women <50 years treated between 1989 and 1998 (10-year cumulative incidence: 2.9 %, 95 % CI 1.9–4.4 %). For women ≥50 years diagnosed from 1999 to 2004 and treated with mastectomy, the 10-year cumulative incidence was lowest at 0.6 % (95 % CI 0.2–1.2 %).

In a subgroup analysis of women diagnosed with DCIS between 1999 and 2004, the Cox model including grade was comparable to the main model (data not shown). The difference in iIBC risk after BCS alone and BCS+RT was of the same magnitude [e.g., for women ≥50 years in the first 5 years after DCIS treatment: HR 4.78, 95 % CI 2.64–8.65 (model including grade) vs HR 4.57, 95 % CI 2.55–8.22 (main model)]. Additionally, iIBC risk did not differ by grade (adjusted estimate for intermediate vs low grade and high vs low grade: HR 1.25, 95 % CI 0.80–1.97 and HR 1.19, 95 % CI 0.75–1.87, respectively).

### Contralateral invasive breast cancer

Contralateral IBC occurred in 536 women. The median time to cIBC was 6.2 years (interquartile range 3.3–9.8 years). Cumulative incidences of cIBC at 15 and 20 years after DCIS diagnosis were 6.4 % (95 % CI 5.9–7.1 %) and 8.9 % (95 % CI 7.7–10.1 %), respectively, reaching a rate of 0.4–0.5 % per annum. The risk of cIBC did not differ by treatment, period of diagnosis, or age group (see Supplemental Table 1, which demonstrates the multivariate Cox proportional hazards analysis for cIBC risk).

**Fig. 4:**
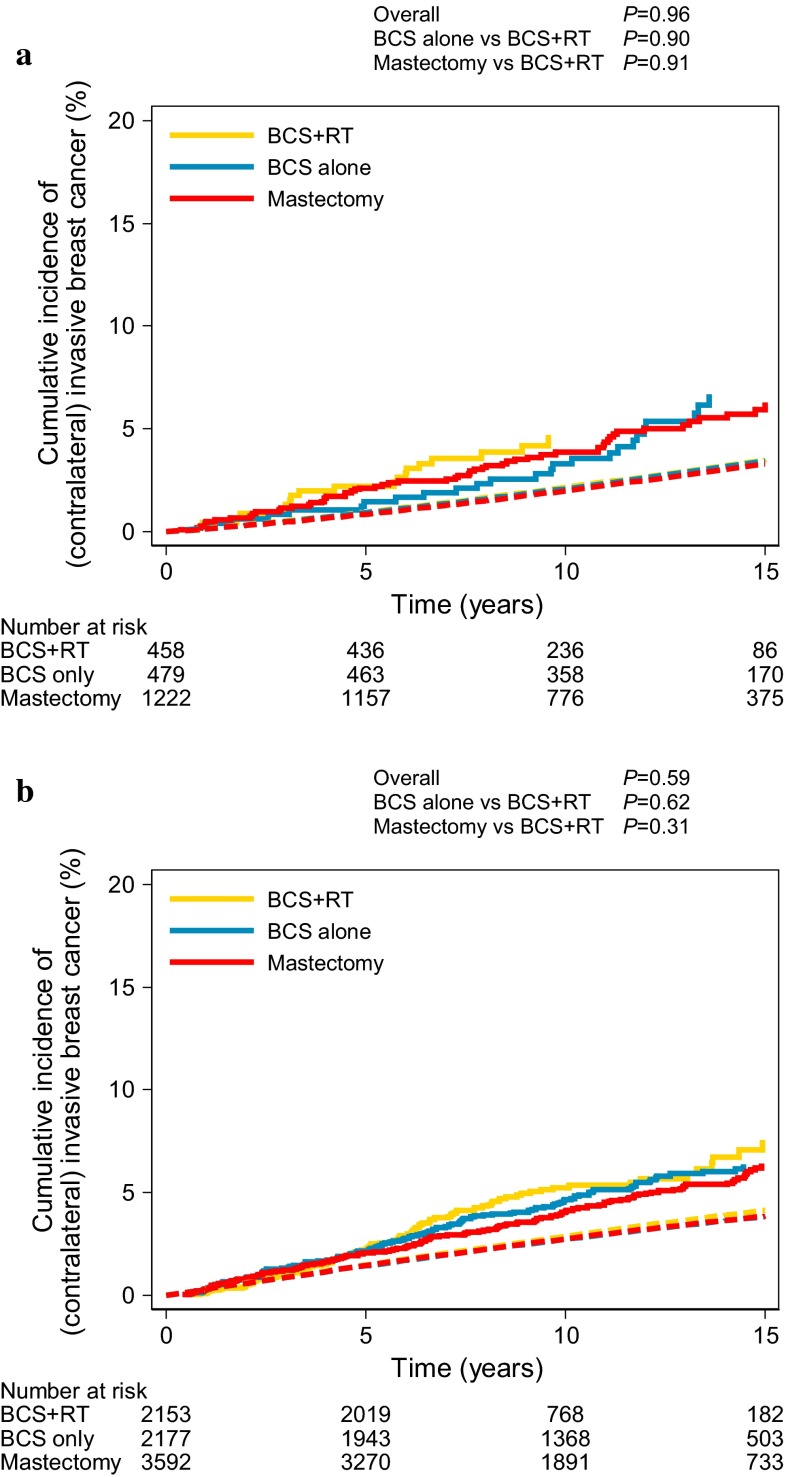
Cumulative incidence of cIBC by treatment strategy compared with the expected cumulative incidence of IBC in the general population (*dashed line*) for **a** women <50 years, and **b** women ≥50 years, with death as competing risk. *BCS* breast-conserving surgery, *RT* radiotherapy. *P* values based on competing risk regression, adjusted for age (continuous) [30]

The cumulative risk of cIBC is visualized in Fig. 4. The absolute risk of developing cIBC in women treated for DCIS was slightly higher than the risk of IBC in the general population (3.4 % at 15 years).

## Discussion

To the best of our knowledge, this is the largest population-based, nationwide cohort study with accurate and complete long-term outcome data of subsequent invasive breast cancer after DCIS treatment. For women treated with BCS, our study confirms the protective effect of RT with regard to iIBC risk shown by randomized controlled trials (RCTs) [23–27, 32]. Importantly, the benefit of RT regarding iIBC risk may differ by age and follow-up interval. It appeared that the use of RT after BCS in women <50 years reduced the risk of iIBC only in the first years after treatment. In women ≥50 years, iIBC risk remained increased during subsequent follow-up after BCS alone, compared to BCS+RT, but the difference became less pronounced with longer follow-up. Our results suggest that RT is effective in treating microscopic residual disease, but may not prevent *de novo* IBC in DCIS patients. One of the RCTs also found that the beneficial effect of RT seemed to be restricted to the first 5 years after treatment [24].

Interestingly, the results of our Cox regression analysis point towards less benefit from RT in women <50 years than in older women. This observation could be due to confounding if for example younger women treated with RT were more likely to have DCIS with unfavorable prognostic features. However, a meta-analysis of the RCTs also found age to modify the benefit of RT: women <50 years showed a smaller proportional risk reduction in the rate of local recurrence (either in situ or invasive) than women ≥50. A trend in the proportional reduction with age was also found when the data were subdivided into five age groups and was independent of histological grade, comedonecrosis, nuclear grade, or architecture [32].

Additionally, we found high iIBC risks after BCS—either with or without RT—in women <50 years. Moreover, these young women treated with mastectomy had a higher cumulative iIBC incidence than older women who received this treatment. Prior studies have also reported that local recurrences following mastectomy seem to occur particularly in younger women [33–35]. Data that may explain this higher risk in younger women are limited and inconsistent [35–38]. Despite the increased iIBC risk, young age per se should not be considered a contraindication for BCS, especially because breast cancer-specific mortality has not been shown to differ between mastectomy and BCS [32, 39].

Another clinical relevant observation is that the absolute risk of cIBC was low with a rate of 0.4–0.5 % per annum. This result is comparable to the population-based study by Falk et al. (*n* = 3,163; median follow-up 5.2 years) [15]. Despite the low cIBC risk, a marked increase in the use of contralateral prophylactic mastectomies among women with DCIS in recent years has been reported [40–42]. Because contralateral prophylactic mastectomies will not likely result in any survival advantage despite the minimization of cIBC risk [43] and are not risk-free [43–45], we advocate that prophylactic contralateral mastectomies for DCIS in women without hereditary breast cancer risk should be discouraged.

One of the strengths of our study was that we differentiated between invasive and non-invasive recurrences. Our 10-year estimates are in line with the 10-year absolute risks reported in other population-based cohort studies and RCTs [15, 17, 32]. However, direct comparison with previous studies, which focused most of their analyses on any local recurrence as outcome, is often difficult. Differences in study design, inclusion criteria, and statistical methods (e.g., cumulative incidence vs Kaplan–Meier estimates) may for example play a role.

Interestingly, the 10-year cumulative incidence and Kaplan–Meier estimates in two, rather small, North American non-randomized prospective studies of women with “favorable” DCIS treated with BCS alone between 1995 and 2002, were only slightly lower than the 10-year cumulative incidence of iIBC for women diagnosed between 1999 and 2004 and treated with BCS alone in our population-based cohort [21, 22]. On the other hand, the estimated 7-year iIBC cumulative incidences in a fifth RCT between BCS+RT (*n* = 287) and BCS alone (*n* = 298) in a selected “good-risk” group of women were much lower [23]. Notably, in this RCT in which 62 % of women used tamoxifen, only eight iIBCs occurred in the BCS-alone arm, and only one in the BCS+RT arm (median follow-up 7.2 years). The differences in risk estimates could be explained by differences in selection criteria, and utilization of tamoxifen, although the effect of tamoxifen on iIBC seems to be minimal [46].

A limitation of our study is the potential of confounding by indication. As the allocation of DCIS treatment was not randomized and the indication for treatment may have been related to the risk of IBC, this could have introduced bias. It is plausible to assume that women with less favorable characteristics more often received adjuvant RT after BCS. Therefore, if confounding by indication plays a role, this will probably have resulted in an underestimation of the difference in iIBC risk between BCS+RT and BCS alone. Although grade was associated with treatment strategy in our study, we found that grade was not a confounding factor in our subgroup analysis, as grade was not associated with iIBC risk. We did not have information on several other risk factors associated with local recurrence, such as DCIS size and margin status after excision. However, it is still uncertain to what extent these factors are associated with subsequent invasive breast cancer risk [47, 48] and therefore whether these could be confounding factors in our study.

A last issue concerns the applicability of our results to today’s clinical practice. Our study shows that the risk of developing iIBC was lower for women diagnosed between 1999 and 2004 than for women diagnosed between 1989 and 1998, while risk of cIBC was similar for both periods. The decrease in iIBC risk over the years was independent of treatment strategy and is likely the result of the detection of relatively more harmless DCIS lesions and improvements in preoperative assessment and surgical management. Most likely, the risk found for the latter period reflects the upper boundary of today’s risk of iIBC in women treated for DCIS, as patient evaluation and selection for treatment have evolved further since 2004.

It should be emphasized that the women in our cohort were not treated with tamoxifen for DCIS. In the Netherlands, hormonal treatment for DCIS is not recommended and its use is very limited in current clinical practice [49, 50]. A meta-analysis of RCTs assessing the effect of postoperative tamoxifen showed a reduced rate of cIBC, but no impact on the risk of iIBC or all-cause mortality [46]. The difference in absolute IBC risk between our cohort and a population in which tamoxifen was more common will therefore probably be limited.

In summary, our finding that the reduction in iIBC risk among women treated with BCS + RT, compared to BCS alone, diminishes with longer follow-up, emphasizes the importance of clinical studies with long-term follow-up. Furthermore, the beneficial effect of RT seems to be smaller among younger women and should be investigated further. Finally, the low risk of cIBC does not justify contralateral prophylactic mastectomies for many women with unilateral DCIS.

## Electronic supplementary material

Below is the link to the electronic supplementary material.

Multivariate cox regression analysis for contralateral invasive breast cancer in women treated for DCIS^a^ (DOCX 13 kb)

## Abbreviations

BCS: Breast-conserving surgery
CI: Confidence interval
cIBC: Contralateral invasive breast cancer
DCIS: Ductal carcinoma in situ
HR: Hazard ratio
IBC: Invasive breast cancer
iIBC: Ipsilateral invasive breast cancer
NCR: Netherlands cancer registry
PALGA: Nationwide network and registry of histology and cytopathology, the Netherlands
RCT: Randomized controlled trial
RT: Radiotherapy
