# Supplementary material for: Subsequent risk of ipsilateral and contralateral invasive breast cancer after treatment for ductal carcinoma in situ: incidence and the effect of radiotherapy in a population-based cohort of 10,090 women
Source: Breast Cancer Res Treat. 2016 Sep 8;159(3):553–63. doi: 10.1007/s10549-016-3973-y (PMC5021731; doi:10.1007/s10549-016-3973-y)
Supplement: Supplementary file 1 — Multivariate cox regression analysis for contralateral invasive breast cancer in women treated for DCISa (DOCX 13 kb) [file 10549_2016_3973_MOESM1_ESM.docx]

**Supplemental table 1. Multivariate Cox regression analysis for contralateral invasive breast cancer in women treated for DCIS^a^**

|  | **Total cIBC** | **Person-time, years** | **HR (95% CI)** | ***P*-value** |
| --- | --- | --- | --- | --- |
| **Treatment** |  |  |  |  |
| BCS + RT | 126 | 24382 | ref |  |
| BCS alone | 147 | 27910 | 1.05 (0.82 - 1.34) | 0.72 |
| Mastectomy | 263 | 57324 | 0.91 (0.73 - 1.13) | 0.38 |
| **Age group at DCIS diagnosis** |  |  |  |  |
| <50 years | 119 | 26210 | ref |  |
| ≥50 years | 417 | 83405 | 1.10 (0.78-1.54) | 0.59 |
| **Period of DCIS diagnosis** |  |  |  |  |
| 1989 - 1998 | 320 | 67914 | ref |  |
| 1999 - 2004 | 216 | 41702 | 1.17 (0.97 - 1.42) | 0.10 |
| **Follow-up interval** |  |  |  |  |
| 0-5 years | 211 | 48621 | ref |  |
| 5-10 years | 200 | 37745 | 1.25 (1.02-1.53) | 0.03 |
| ≥10 years | 125 | 23249 | 1.37 (1.05-1.79) | 0.02 |

^a^ With age as primary time-scale and treatment as time-varying variable.

cIBC = contralateral invasive breast cancer; HR = hazard ratio; CI = confidence interval; BCS = breast-conserving surgery; RT = radiotherapy.
